# Micronucleus cytome assay in the differential assessment of cytotoxicity and genotoxicity of cadmium and lead in *Amietophrynus regularis*

**DOI:** 10.17179/excli2017-887

**Published:** 2018-01-11

**Authors:** C.G. Alimba, A.M. Aladeyelu, I.A. Nwabisi, A.A. Bakare

**Affiliations:** 1 Cell Biology and Genetics Unit, Department of Zoology, University of Ibadan, Nigeria

**Keywords:** acute toxicity, amphibian decline, cadmium and lead compounds, cytotoxicity, genome instability, micronucleus cytome assay, toad

## Abstract

Amphibians are increasingly being used as bio-indicator of contamination in ecosystems due to their sensitivity to xenobiotics in the environment. Cadmium and lead compounds, ubiquitous mutagens and carcinogens, are capable of eliciting genome instability in adult toads which may enhance amphibian decline. Micronucleus cytome (MN-cyt) assay, a comprehensive cytogenetic test for the assessment of genome instability induced by xenobiotics in organisms, was utilized in the differential cytogenotoxic evaluation of Cd and Pb in adult *Amietophrynus regularis*. *A*. *regularis *was exposed to six concentrations (8 - 512 mg/L) of the metal solutions to determine 96 h acute toxicity. Four toads per group were exposed to five sub-lethal concentrations (5 - 75 %) of the 96 h LC_50_ of the metals for 14 days. At post exposure, bone marrow and peripheral erythrocytes were collected for MN-cyt analysis. The metals induced differential concentration and time-dependent increase in mortality with 96 h LC_50_ of 36.36 mg/L (Cd) and 112.06 mg/L (Pb). No observable effective concentrations (NOEC); Cd=8 and Pb=32 (mg/L) and Lowest observable effective concentrations (LOEC); Cd=16 and Pb=64 (mg/L) were recorded for the metals. Derived toxicity factor (TF) showed that Cd was 3.08 times more toxic to the toads than Pb. The metal solutions induced significant (*p*<0.05) increase in frequencies of MN, binucleated, nuclear bud, notch, lobe, vacuolated erythrocytes, apoptosis and necrosis compared to the negative control. Cd elicited 1.42 and 3.26 folds increase in MN and NAs respectively, than Pb. MN-cyt assay is a suitable cytogenetic tool for assessing genome instability in *A. regularis*. Increased genetic instability induced by Cd and Pb may be associated with genetic related syndromes; neoplasms, reproductive dysfunctions and mortality. This suggests threat to amphibian health and may enhance population decline.

## Introduction

Decline in amphibian biodiversity is one among the challenges currently facing conservationists worldwide. A global comprehensive assessment of amphibian population decline showed that about 32.5 % amphibians were classified as vulnerable, endangered or critically endangered, 7.4 % species as critically endangered and 43 % experiencing different forms of decline (Stuart et al., 2004[59]). This classification elicited numerous studies geared towards understanding the possible causes and mechanisms involved in amphibian decline. The outcome increased awareness among herpetologists on the possible role(s) of habitat destruction and exposure to harmful chemicals (Blaustein et al., 2003[10]; Lips et al., 2005[40]), which may have increased morbidity and mortality among exposed populations. For instance, the possible role(s) of pesticides, herbicides and fungicides on developmental deformities and survival of amphibians were investigated for over a decade as the major cause of amphibian decline (Berrill et al., 1998[9]; Brunelli et al., 2009[12]). Also, the impact of toxic metals on amphibian health and mortality which may lead to decline was also considered among the harmful chemicals. However, available reports showed the harmful effects of these metals on mortality, acute toxicity, developmental abnormalities and genotoxicity in the embryonic/larva stage of this group of vertebrate that exhibit quasi-terrestrial life style (Rosenberg et al., 2003[50]; Mouchet et al., 2007[45]; Patar et al., 2016[49]). There is paucity of information on the impact of metals on genomic instability in adult anurans (Rosenberg et al., 2003[50]; Said et al., 2016[52]). It is noteworthy that unlike pesticides, fungicides and herbicides with short half-life and which are synthesized to kill or cause morbidity to specific group of organisms, metals readily accumulate in the body organs and are rarely biodegraded. These ubiquitous carcinogens, mutagens and endocrine disruptors are capable of inducing cytogenetic, reproductive and systemic abnormalities in all biological forms with little or no species specificity (Patar et al., 2016[49]). 

Adult anurans are rarely used in cytogenotoxicological studies despites their suitability as bioindicator for monitoring environmental contamination, and wide range of adaptations to wetlands and landscape (US EPA, 2002[61]). Moreover, they live greater part of their lives on moist terrestrial habitats and are important in the ecosystem population dynamics. Adult anurans are exposed to toxic metallic compounds via absorption through the permeable skin during dermal contact with water, sediments and soil or hibernation in soils, and ingestion of food (SEAC, 1996[53]). Inadequate information exists on the use of adult toads as bioindicator of genome instability assessment of heavy metals. Despites that genome instability and cytotoxicity have been increasingly associated with poor fecundity and reproductive fitness, decreased cell survival, developmental abnormalities, change in genetic population dynamics and tumor neoplasm (Malins et al., 1988[42]; Shugart, 2000[57]; Andreassi et al., 2011[4]). These are important factors to be considered in relation to amphibian decline. 

Cadmium (Cd) and lead (Pb) are common deleterious metals that readily bioaccumulate in biological systems from different environmental matrices. They are without any known useful physiological functions even at trace concentrations. With the persistence of these metals in the ecosystems, they exert numerous adverse effects on wildlife and humans. These known carcinogens and mutagens are readily transported to the bone marrow, kidney and liver where they distort the haematopoietic system (Rosenberg et al., 2003[50]). The study herein reports for the first time the cytotoxicity and genotoxicity of Cd and Pb in the highly differentiating bone marrow and peripheral erythrocytes of adult toads using micronucleus cytome assay (MN-cyt). Micronucleus assay (MN) is a frequently used cytogenetic test for the assessment of structural and numerical chromosomal aberrations induced by clastogens and aneugens, due to its suitability, reproducibility and cost effectiveness in lower vertebrates (Mouchet et al., 2007[45]; Alimba and Bakare, 2016[2]). It was utilized for the first time to assess the cytogenetic damage induced by genotoxins present in polluted fresh water on peripheral blood erythrocytes of Newt (*Pleurodeles waltl*) (Jaylet et al., 1986[36]). However, anuran MN cytome assay has not been previously utilized in the genotoxic and cytotoxic assessments of Cd and Pb in heamotopoietic bone marrow system of the adult toads. 

Micronucleus cytome assay is a comprehensive system for assessing DNA damage and cytotoxicity at the chromosomal level using the biomarkers: micronuclei (MN; biomarker of chromosome breakage and/or loss), nuclear buds (Nbud; biomarker of gene amplification and/or elimination of DNA repair complexes) and nuclear abnormalities (NAs; Lobe and Notch). Cytotoxicity is measured via frequencies of necrotic, apoptotic and binucleated cells. Its detailed protocol was first described in *in vitro* test system by Fenech (2007[24]) and later described in invertebrate (Bivalve, genus: *Mytilus* by Bolognesi and Fenech (2012[11]). However, its use in lower vertebrates to assess genome instability is scarce. The study herein reports the acute toxicity (96 h LC_50_), a recommended static bioassay for the preliminary toxicological assessment of chemicals in biological test systems (ASTM, 2002[7]; US EPA, 2005[62]), and genome instability elicited by sub-lethal concentrations of Cd and Pb in bone marrow and peripheral blood erythrocytes of adult *Amietophrynus regularis*. 

## Materials and Methods

### Metal compounds

Cadmium (II) chloride (CdCl_2_) and lead (II) nitrate Pb(NO_3_) of analytical grades (Sigma, St. Louis, MO, USA) were used as the source of metals.

### Animals

Adult toads, *A. regularis*, collected from the Zoological garden, University of Ibadan, Nigeria were utilized. They were housed in plastic cages with perforated lids, containing dechlorinated tap-water and acclimatized for 14 days in the Animal house, Department of Zoology, University of Ibadan. They were maintained in laboratory conditions of 12 h dark and light photoperiod and temperature of 26±9 °C. The water was renewed every 48 h throughout the experimental duration and they were fed with earthworms (*Eudrilus eugeniae*). 

### Analysis of acute and sub-lethal toxicity of Cd and Pb 

Following range finding test for the metals, ten *A. regularis* with body weight range of 117.4 - 131.9 g and mean body weight of 128.34±2.05 g, were randomly distributed into 0, 8, 16, 32, 64, 128, 256 and 512 (mg/L) of Cd and Pb in 25 L plastic tanks to determine the 96 h acute toxicity (LC_50_) and behavioural changes in accordance with ASTM (2002[7]) and Arrieta et al. (2000[5]). Safe concentrations of the metals at 96 h were obtained by multiplying the 96 h LC_50_ by a factor of 0.1 in accordance with EIFAC (1998[20]). No Observed Effective Concentration (NOEC), Low Observed Effective Concentration (LOEC) and Toxicity Factors (TF) of the metals were also determined in accordance with Sadeghi and Imanpoor (2015[51]). Metal solutions were prepared immediately before use and toads were not fed during the acute toxicity study. Mortality was determine by visual observation and recorded for every 24 h. Toads were considered dead when no movement was observed after gentle prodding with a glass rod. For the sub-lethal toxicity assessment, four *A. regularis* per group were randomly distributed into 25 L tanks containing 5, 10, 25, 50 and 75 % of the sub-lethal concentrations (which correspond to 1.82, 3.64, 9.09, 18.18 and 27.27 mg/L for Cd, and 5.60, 11.21, 28.02, 56.03 and 84.05 mg/L for Pb, of the 96 h LC_50_ for the metals) were selected in accordance with Nikoloff et al. (2014[47]), for 14 days. Similar treatment was given to toads in 0.01 mL/L Benzene, a haematopoietic genotoxic inducer (IPCS, 1993[33]; WHO, 2003[63]), and dechlorinated tap water as positive and negative controls respectively.

### Micronucleus cytome assay

At the end of exposure, toads were anesthetized by immersing in ice water in accordance with American Society of Ichthyologists and Herpetologists (ASIH, 2004[6]) criteria, and then sacrifice by double pithed. Peripheral blood was collected into EDTA bottles using heparinized syringe via cardiac puncture after which the toads were dissected and femoral bones collected into vials containing 0.9 % buffered normal saline. Cells were then aspirated from both femurs using 0.5 mL of 2.2 % sodium citrate into Eppendorf tubes. Aspirated cells were centrifuged at 2000 rpm for 10 min and the supernatant decanted. Smears of cell suspension were prepared on three pre-cleaned and grease free microscope slides per toad. Similarly, thin smears of the peripheral blood erythrocytes were prepared on three pre-clean and grease free microscope slides per toad. The prepared slides were allowed to air dry for 24 h, fixed in absolute (100 %, v/v) cold methanol (4 °C) for 20 min and counter stained with 5 % Giemsa and May-Grunwald stains for 10 min. 2000 cells per slide were analyzed to score for MN, nuclear abnormalities (NAs), necrosis and apoptosis in accordance with standard protocols (Fenech, 2007[24]; Alimba and Bakare, 2016[2]). 

### Statistical analysis 

Data obtained from the acute toxicity (mortality) were analyzed using probit analysis (Finney, 1971[26]) with SPSS™ version 17.0 and presented as LC_5 _(lethal concentration that produced 5 % mortality), LC_50_ (lethal concentrations that produced 50 % mortality), and LC_95_ (lethal concentration that produced 95 % mortality) at the corresponding 95 % confidence intervals. Also, TF for 24 hourly relative potency measurements for the metals were determined. Data for the MN, NAs, necrosis and apoptosis are presented as mean ± SE (standard error). Significant difference among the various treatment and control groups was determined using one-way ANOVA, while Dunnett's multiple post-hoc test was used to compare the level of significance (*p*< 0.05) of each treatment group with the negative control.

## Results

**Figure 1:**
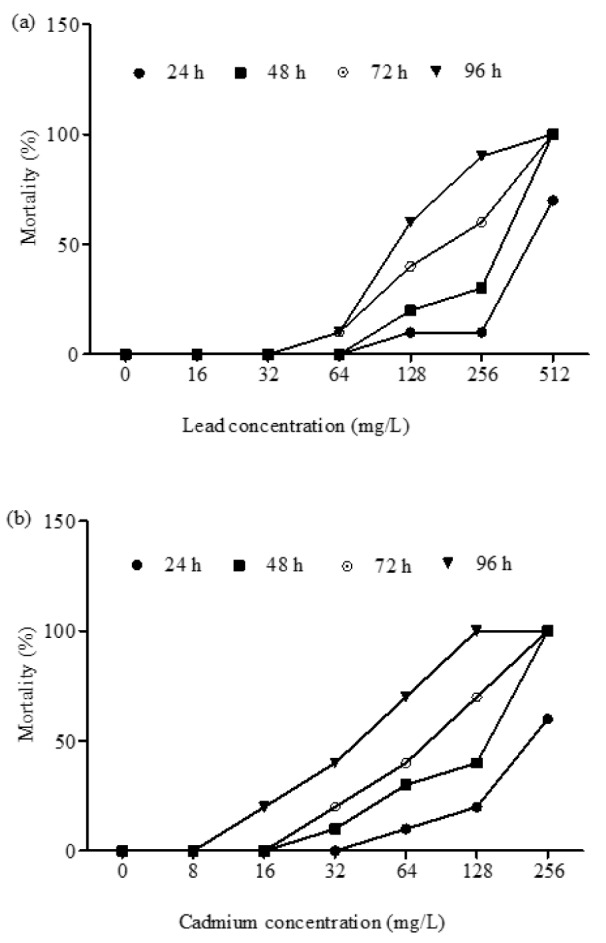
Figure 1a, b: Percentage mortality of *Amietophrynus regularis* after 96 h exposure to different concentrations of lead (II) nitrate (1a) and cadmium (II) chloride (1b).

There was concentration dependent increase in the mortality of Cd and Pb exposed toads. All toads exposed to 128 and 256 mg/L of Cd and 512 mg/L of Pb died (100 % mortality). There was no mortality at 8 mg/L of Cd, and between 8 and 32 mg/L of Pb during exposure (Figure 1a, b(Fig. 1)). The derived toxicity indices from the concentration-mortality data (Figure 1a, b(Fig. 1)) showed that NOEC for Cd and Pb are 8 and 32 mg/L respectively, while the LOEC for Cd and Pb are 16 and 64 mg/L respectively. Also, the safe concentrations for Cd and Pb were 3.64 and 11.21 mg/L respectively. The hourly derived LC_5_, LC_50_ and LC_95_ values for both Cd and Pb acute toxicity showed that *A. regularies* mortality increased with metal concentrations and exposure duration. The 96 h LC_50_ determined for Cd is 36.36 mg/L, and 112.06 mg/L for Pb (Tables 1(Tab. 1), 2(Tab. 2)). The computed TF (3.08) for both metals showed that Cd was 3 folds highly toxic to *A. regularis* compared to Pb. Also, the clinical signs of metal induced toxicity observed in the exposed toads which include avoiding contact with the metal solutions by climbing on one another, inactivity and peeling of skins, were severe among Cd treated toads and increased according to concentrations of the metals.

**Figure 2:**
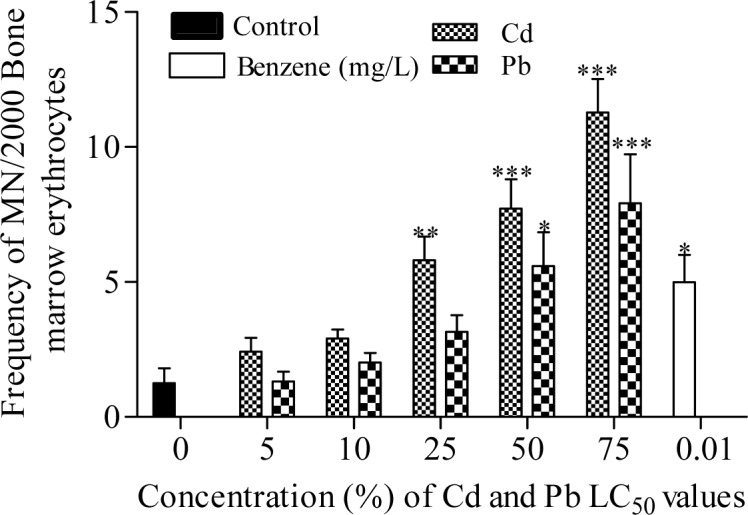
Frequency of micronuclei induction in bone marrow erythrocytes of *Amietophrynus regularis* following exposure to different concentrations of lead (II) nitrate and cadmium (II) chloride. End point represents mean ± SE per treatment group containing four toads. Values are significantly different from the negative control as identified by Dunnett Multiple posthoc test (*p<0.05, **p<0.01, ***p<0.001).

**Figure 3:**
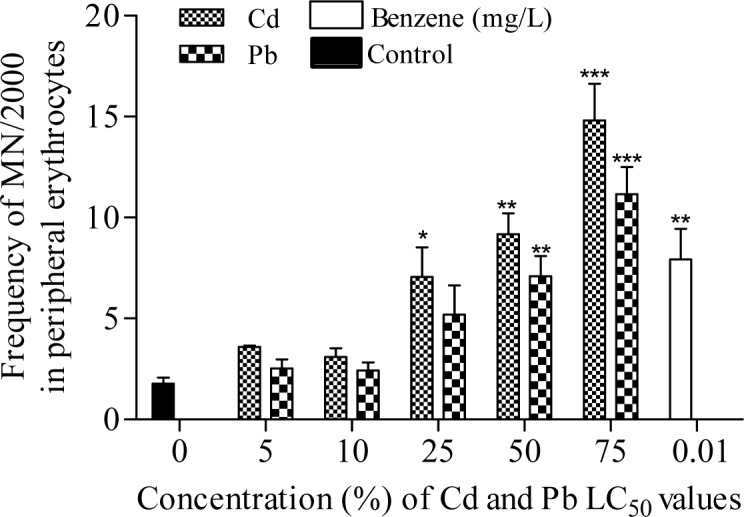
Frequency of micronuclei induction in peripheral blood erythrocytes of *Amietophrynus regularis* following exposure to different concentrations of lead (II) nitrate and cadmium (II) chloride. End point represents mean ± SE per treatment group containing four toads. Values are significantly different from the negative control as identified by Dunnett Multiple posthoc test (*p<0.05, **p<0.01, ***p<0.001).

**Figure 4:**
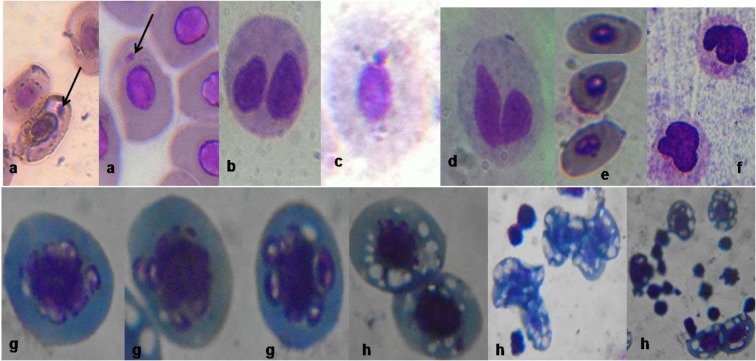
Erythrocytes scored in the MN-cyt assay: (a) micronucleated cells, (b) binucleated cell, (c) nuclear buded cell, (d) notch nucleated cells, (e) vacuolated nucleated cells, (f) lobe nucleated cells, (g) different apoptotic stages and (h) different necrotic stages in *Amietophrynus regularis* exposed to different concentrations of Cd(II) and Pb(II) salts (Magnification: x1000).

**Figure 5:**
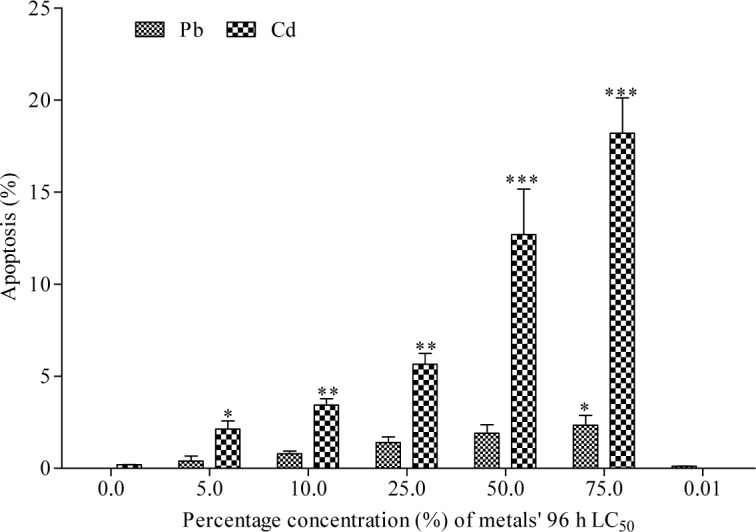
Effect of Cd and Pb treatments on the percentage of apoptosis in peripheral blood erythrocytes of *Amietophrynus regularis*. End point represents percentage of mean ± SE per treatment group containing four toads. Values are significantly different from the negative control as determined by Dunnett Multiple posthoc test (*p<0.05, **p<0.01, ***p<0.001).

**Figure 6:**
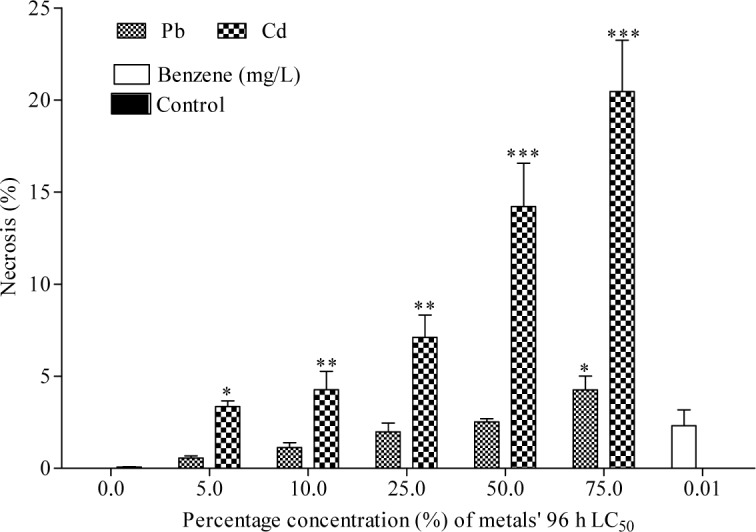
Effect of Cd and Pb treatments on the percentage of necrosis in peripheral blood erythrocytes of *Amietophrynus regularis*. End point represents percentage of mean ± SE per treatment group containing four toads. Values are significantly different from the negative control as determined by Dunnett Multiple posthoc test (*p<0.05, **p<0.01, ***p<0.001).

The frequency of MN induced in bone marrow erythrocytes of Cd exposed toads was highly significant (*p*<0.0001; r=0.69) with 1.93, 2.31, 4.63, 6.16 and 9.00 folds (corresponding to 5, 10, 25, 50 and 75 % of Cd concentrations respectively) increase than the negative control (Figure 2(Fig. 2)). Similarly, Pb significantly (*p*=0.0005; r=0.40) increased MN formation in bone marrow erythrocytes by 1.05, 1.61, 2.52, 4.47 and 6.32 folds (corresponding to 5, 10, 25, 50 and 75 % of Pb concentrations respectively) compared to the negative control. Benzene (0.01 mL/L), a known haematopoitic dysfunction and chromosome aberration inducing chemical (IPCS, 1993[33]) used as positive control, induced 3.98 fold increase in MN compared to the negative control. Fold increase of MN induced by benzene is within 25 % and 50 % concentrations of Cd and Pb respectively. There was similarity in the trend of MN frequencies induced by Cd (*p*<0.0001; r=0.66 with fold increase; 1.78, 1.81, 3.99, 5.19 and 8.36 (corresponding to 5, 10, 25, 50 and 75 % of Cd) and Pb (*p*<0.0001; r=0.57 with fold increase; 1.43, 1.37, 2.94, 4.01 and 6.31 (corresponding to 5, 10, 25, 50 and 75 % of Pb) in the peripheral blood erythrocytes compared to the negative control (Figure 3(Fig. 3)). Both Cd and Pb increased NAs in peripheral blood erythrocytes of treated toads compared to the negative control (Table 3(Tab. 3)). The scored NAs which include binucleated blood erythrocytes (Figure 4b(Fig. 4)), nuclear bud (Figure 4c(Fig. 4)), notch nucleus (Figure 4d(Fig. 4)), vacuolated nucleus (Figure 4e(Fig. 4)) and lobe nucleus (Figure 4f(Fig. 4)) were significantly (*p*<0.05) higher than the control except for vacuolated nucleus. There was concentration-dependent significant increase in the percentage apoptotic erythrocytes (Figure 4g(Fig. 4)) induced by Cd (*p*<0.0001; r=0.68) and Pb (*p*<0.016; r=0.29) (Figure 5(Fig. 5)), and necrotic erythrocytes (Figure 4h(Fig. 4)) induced by Cd (*p*<0.0001; r=0.72) and Pb (*p*<0.0009; r=0.41) in peripheral blood erythrocytes compared to the negative control (Figure 6(Fig. 6)). 

## Discussion

Increasing metal contamination of agricultural and forest land, and waterways where anurans readily inhabit, due to fertilizer application, industrial and mining activities, may greatly impact on amphibians' health. Cd and Pb were selected for this study due to their environmental relevant concentrations which have increased morbidity and mortality in humans, with scanty information on wildlife populations. For instance, in 2009, large-scale epidemics of Pb poisoning involving more than 2000 children living near smelting plants and sparking riots was reported in China (Li et al., 2016[39]). Also in 2010, massive deaths of over 200 children with non-specific symptoms reported in Northern Nigeria was associated with outbreak of acute lead poisoning due to illegal mining of gold-rich ore (Lo et al., 2012[41]). Cadmium poisoning that led to the deaths of over 100 people who suffered Itai-itai disease between 1910 and 2007 after consuming contaminated rice grown in Jinzu River basin in Toyama, Japan (Matsunami, 2010[44]), showed the level of deleterious effects of these metals. Considering that Cd and Pb constitute top prioritized hazardous substances (IPCS, 1992[34]; DeRosa et al., 1996[19]), there is scarcity of information on the genotoxicity and cytotoxicity impacts of these metals on native adult toads residing in Cd and Pb polluted sites. However, the study herein utilized a standard laboratory simulated experiment as a preliminary study, to understand the possible acute toxicity and cytogenotoxicity induced by Cd and Pb in adult toads. 

The 96 h LC_50_ of Cd showed that Cd was three fold more harmful to the exposed toads than Pb. This suggests that *A. regularis* tolerated the deleterious impacts of Pb than Cd. Concentration-dependent increase in mortality induced by Cd and Pb in the exposed toads may be related to the rapid bioaccumulation of the metals in the tissues through the permeable skin (Arrieta et al., 2000[5]; Said et al., 2016[52]). Behavioral changes observed during exposure to the metals, which include climbing on one another was to avoid contact with the metal solution so as to prevent metal absorption and mortality. Although, it is not clear why there was peeling of the toad skin during exposure to the metals, it may be linked to physiological behavior enhancing metal speciation and or sequestration via excretion after redistribution of the metals to less sensitive target sites like the skin (Hopkins, 2007[30]; Said et al., 2016[52]). Similar differential acute toxicities of Cd and Pb were observed in *Bufo maculatus* (Enuneku and Ezemonye, 2012[23]). Previous studies had also shown that differential toxicity of Cd and Pb to anurans were severe to the developmental stages of these animals (Arrieta et al., 2000[5]; Mouchet et al., 2007[45]; Enuneku and Ezemonye, 2012[23]; Patar et al., 2016[49]; Said et al., 2016[52]). The derived safe concentrations of 3.64 mg/L (Cd) and 11.21 mg/L (Pb) for the toads used herein, are significantly lower than 1310 - 1586 mg of Pb per kg of soils observed in farmlands around Pb contaminated goldmine in Northern Nigeria (Abdulkareem et al., 2015[1]), 2000 mg of Pb per kg of soil (Elsokkary et al., 1995[21]) and 400 mg of Cd per kg of aquatic sediments (Elsokkary and Muller, 1990[22]) from Egypt. This may suggest a higher level of morbidity and eventual death of toads and other amphibians that may be inhabiting such metal laden sites. 

Cd and Pb sub-lethal concentrations used in this study were within the safe concentration limits, NOEC and LOEC values determined for the *A. regularis*. Although, these selected concentrations did not induce mortality of the exposed toads even at 14 days of exposure, they however elicited myriad of genotoxic and cytotoxic effects. Significant increase in the frequencies of micronucleated, apoptotic, nuclear bud, necrotic, binucleated, vacuolated, notch and lobe nucleated erythrocytes (Figure 4a-h(Fig. 4)) formed in the bone marrow and peripheral blood erythrocytes of the exposed toads to sub-lethal concentrations of Cd and Pb suggests that these metals are clastogenic and/or aneugenic to dividing cells in the bone marrow of the toads. These metals possibly interfered with DNA repair systems (Calsou et al., 1996[13]; Hartwig, 1998[29]), induced DNA strand breaks and/or mitotic spindle dysfunction (Snyder, 1988[58]; Seoane and Dulout, 2001[54]), and generated reactive oxygen species and glutathione depletion (O'Brien and Salacinski, 1998[48]) in the haematopoietic system (bone marrow) of the toads resulting in the observed cytogenotoxic biomarkers (Figure 4(Fig. 4)). The observed differential alterations in genotoxic and cytotoxic biomarkers induced by the investigated metals herein, indicate that Pb is a weaker genotoxin and cytotoxin than Cd. This is in agreement with its (Pb) being classified as probable human carcinogen (Group 2B) (IARC, 2006[31]) and Cd as carcinogen (Group A) (IARC, 1993[32]). 

Significant increase in the frequency of MN in the treated toads suggests that the metals induced perturbation in the heamatopoiesis of the treated toads which resulted in acentric chromosome fragmentation, acentric chromatid fragmentation or whole chromosome loss that were not included in the daughter nuclei after telophase during cell division (Mateuca et al., 2006[43]). It is also possible that the metals induced perturbation in the heamatopoiesis of the treated toads which increased gene amplification and were localized to the periphery of the nucleus during S phase of the cell cycle (Shimizu et al., 1998[56]). This may account for the significant increase in nuclear bud recorded in the exposed toads than the control. Increase in notch, vacuolated and lobe nuclei recorded in the metal treated toads is related to chromosome aneuploidy which probably originated from tubulin failure and terminal acentric fragmentation due to disturbance of the chromatin materials, hence various nuclear evaginations (Fenech et al., 2011[25]). The various abnormal nucleated erythrocytes were scored to complement MN frequency in the genotoxicity assessment considering that they were significantly found in the metal exposed toads compared to the negative control. Moreover, they have been frequently observed in cancer cells and chromosomal unstable cells (Gisselsson et al., 2001[28]; Caruso et al., 2008[14]). 

Increase in the frequency of binucleated erythrocytes suggests blockage of cytokinesis of the dividing cells during erythropoietic process. Increase in the occurrence of apoptotic erythrocytes suggests a self defence mechanism by the toads to eliminate cells with highly damage nuclei by activating intrinsic suicide mechanisms that ultimately destroys the cell (Fraser and Evan, 1996[27]). While necrotic erythrocytes suggest pathologically passive death occurring accidentally due to extreme damage or injury induced by the metals. This cell death type is usually accomplined with cell membrane rupture and spilling out of cellular contents to the surrounding cells (Cohen, 1994[17]). It is plausible that Cd and Pb induced p53 protein expression in the treated toads which led to the activation of genes associated with apoptotic cell formation (Leach, 1998[38]). It is also possible that the metals induced direct genotoxic stress on the bone marrow cells, and the severely damaged cells were eliminated by either programmed cell death (apoptosis) or accidental cell death (necrosis). This is in agreement with the reports that genotoxic stress elicited by cisplastin induced apoptosis in human ovarian carcinoma and human kidney cells was mediated by caspase family proteases (Chen et al., 1999[16]). Also, Cd, As and Pb induced apoptosis in peripheral blood mononuclear cells (lymphoid cells), has been linked to immunotoxicity of the metals (De la Fuente et al., 2002[18]). Increase in binucleated, apoptotic and necrotic erythrocytes in the metal exposed toads showed that the metals are capable of inducing cytotoxic effects in anurans. Studies corroborating the cytotoxic effects of Pb and Cd observed in this study showed that these metals are capable of altering cellular activities via interference with signal transduction pathways which resulted in cell lysis, cellular inflammation, cell death, abnormal cell replication via blockage of DNA repair mechanisms and damage to DNA molecule (Mukharjee et al., 1984[46]; Bae et al., 2001[8]; Chang et al., 2013[15]). Alimba et al. (2016[3]) showed that WIL2-NS lymphoblastoid cells treated with low concentrations of Pb expressed concentration-dependent significant increase in necrotic cells. Also Chang et al. (2013[15]) reported apoptosis in pancreatic β-cells treated with Cd. These *in vitro* studies are in support of the cytotoxicity of Cd and Pb to the exposed toads in this study. 

The results herein showed positive clastogenic effects of Cd and Pb in bone marrow cells of toads. This is in agreement with previous studies wherein low concentrations of Cd salt induced dose-dependent significant increase in MN polychromatic erythrocytes in mice (Jagetia and Adiga, 1994[35]) and DNA damage in human lymphocytes (Shaik et al., 2006[55]). The difference in mortality, cytotoxicity and genotoxicity induced by the metals to treated toads may be attributed to differences in their absorbability, chemical reactivity and complex formation (Bae et al., 2001[8]; Alimba et al., 2016[3]). With the emerging reports that Cd and Pb are capable of inducing DNA damage in anurans (Mouchet et al., 2007[45]; Patar et al., 2016[49]), it is suggested that this may enhance threats to amphibians' health and survival, and their eventual decline. Injuries induced on chromosomes are perhaps the most relevant biological and ecological indicators of adversity. There are associations between cytogenotoxicity outcome and chronic health effects at the population level (Taylor et al., 2005[60]), with cytogenetic damage linked to genetic related syndromes, including neoplasms, physiological and biochemical impairments, reproductive dysfunctions, increase disease susceptibility and reduced adaptive fitness to survival and succession (Kurelec, 1993[37]; Shugart, 2000[57]; Taylor et al., 2005[60]). 

In conclusion, the findings herein showed that Cd and Pb induced differential acute toxicity, MN, binucleated, nuclear bud, bleb, notch and vacuolated erythrocytes, apoptosis and necrosis (biomarkers of genome instability and cytotoxicity) in toads. Whether such effects can be observed *in situ* in Pb and Cd contaminated sites is recommended for investigation to ascertain amphibian health status and survival in the wild. Findings from this study suggest possible roles of toxic metals in anuran genome damage which may enhance amphibian decline.

## Acknowledgements

We thank Mr A. O. Adeleke, technical staff of the Department of Zoology, University of Ibadan, for his assistance in toad collection.

## Conflict of interest

The authors declare no conflict of interest.

## Figures and Tables

**Table 1:**
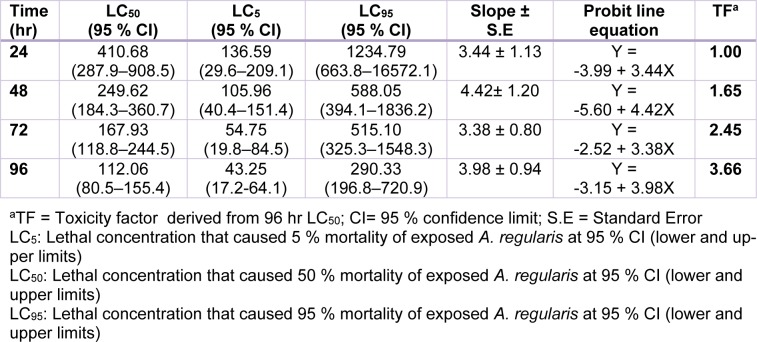
96 h acute toxicity assessment of Pb(II) salt in *Amietophrynus regularis*

**Table 2:**
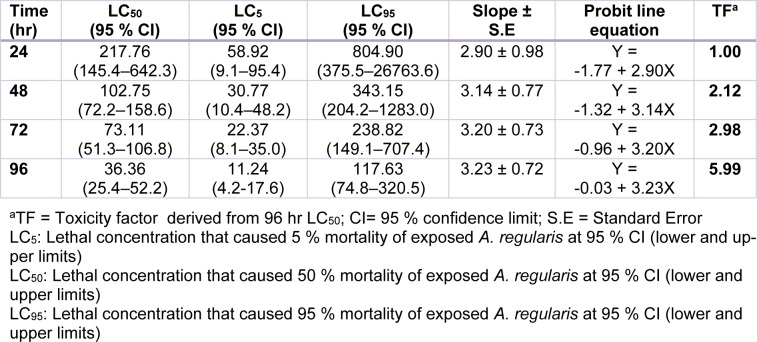
96 h acute toxicity assessment of Cd(II) salt in *Amietophrynus regularis*

**Table 3:**
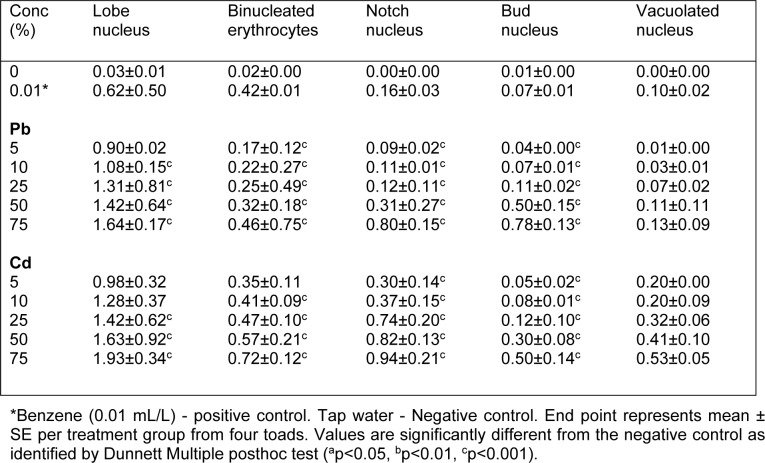
Frequency of nuclear abnormalities in peripheral blood erythrocytes of *Amietophrynus regularis* exposed to sub-lethal concentrations of Cd (II) and Pb (II) salts
